# HDL in Abdominal Aortic Aneurysm: Mechanistic Insight and Therapeutic Potential

**DOI:** 10.1007/s11883-025-01378-9

**Published:** 2026-01-03

**Authors:** Julia C. Catalano, Yanhong Guo, Brandon M. Bordeau, Minzhi Yu, Anna Schwendeman

**Affiliations:** 1 Department of Pharmaceutical Sciences, College of Pharmacy, University of Michigan, Ann Arbor, MI USA; 2 Department of Internal Medicine, Frankel Cardiovascular Center, University of Michigan Medical Center, Ann Arbor, MI USA; 3 Biointerfaces Institute, University of Michigan, Ann Arbor, MI USA

**Keywords:** Abdominal aortic aneurysm, High-density lipoprotein, Apolipoprotein A-1, Vascular smooth muscle cells, HDL therapy

## Abstract

**Purpose of Review:**

This review examines the role of high-density lipoprotein (HDL) in the context of abdominal aortic aneurysm (AAA). It focuses on the correlation between HDL and AAA risk, while also exploring the mechanisms by which HDL may protect against AAA progression.

**Recent Findings:**

Large epidemiological and genetic studies have consistently shown that lower HDL-cholesterol (HDL-C) levels are associated with an increased risk of AAA. HDL exerts a protective effect against AAA formation through its anti-inflammatory, antioxidant, and endothelial protective functions. Recent therapeutic strategies aimed at augmenting HDL functionality, including synthetic HDL (sHDL) and apolipoprotein A-I (ApoA-I) mimetics, have demonstrated promising results in animal models.

**Summary:**

Current evidence supports a protective role for HDL in AAA pathogenesis. Strategies aimed to improve or mimic HDL function represent promising avenues for future AAA treatment, emphasizing the need for continued translational research and clinical development.

## Introduction

Abdominal aortic aneurysm (AAA) is a life-threatening vascular condition characterized by progressive dilation of the infrarenal aorta greater than 3 cm [1]. AAA is typically asymptomatic and often remains undiagnosed until aortic rupture occurs, an event associated with mortality rates of up to 80% [2, 3]. In the United States, AAA affects approximately 6–8% of adults aged 65 years or older [4]. Current management is limited to surgical repair, generally recommended when aneurysm diameter exceeds 5.5 cm or is rapidly expanding, with the remainder advised to monitor size annually [5]. Despite this, it is estimated that 5–10% of patients under surveillance experience AAA rupture before reaching surgery threshold [6].

Dyslipidemia is a recognized risk factor for AAA [7, 8]; however, the specific contributions of lipoproteins to AAA pathogenesis remain incompletely understood. High-density lipoprotein (HDL) exerts multiple vasoprotective functions, including reverse cholesterol transport (RCT), anti-inflammatory, anti-thrombotic, and anti-apoptotic activities. Low plasma HDL concentrations are strongly associated with an increased risk of cardiovascular disease, and recent evidence suggests a relationship between HDL deficiency and the development of AAA [9, 10]. Interventions aimed at raising HDL levels or restoring HDL functionality may offer novel strategies for modulating AAA progression. In this review, we provide an overview of HDL biology in AAA, highlighting epidemiological evidence, protective mechanisms, and functional alterations, and discuss how these may inform HDL-based therapeutic approaches aimed at reducing aneurysm progression and rupture.

## Low HDL is a Risk Factor for AAA

HDL are endogenous anti-atherogenic particles that circulate in the plasma. They comprise a hydrophobic core of cholesterol esters and triglycerides enveloped by an amphipathic layer of phospholipids and unesterified cholesterol [11]. Apolipoprotein A-I (ApoA-I), which accounts for more than 70% of HDL’s protein content, provides structural stability and drives lipid interactions [12]. The primary mechanism underlying HDL’s atheroprotective function is its ability to mediate reverse cholesterol transport (RCT), the process by which HDL removes excess cholesterol from peripheral cells and transports it to the liver for excretion. ApoA-I initiates this process by interacting with the ATP-binding cassette transporter A1 (ABCA1) on macrophages and other peripheral cells. This facilitates the efflux of free cholesterol and phospholipids, forming nascent, discoidal HDL particles. Maturation occurs through the activity of lecithin-cholesterol acyltransferase (LCAT), which esterifies free cholesterol and drives its sequestration into the particle core, forming spherical HDL. Additional cholesterol efflux is mediated by ATP-binding cassette transporter G1 (ABCG1) and scavenger receptor class B type I (SR-BI), both of which preferentially transfer cholesterol to mature HDL particles.

Epidemiological and clinical studies have highlighted an inverse association between HDL and AAA (Table 1). Norrgård et al. were among the first to report that AAA patients have lower serum HDL-cholesterol (HDL-C), observing a nearly 37% and 38% decrease in HDL-C in male and female AAA patients, respectively, compared to healthy controls [13]. In a screening cohort of 1,601 subjects, HDL-C was 16.3% lower in individuals with AAA, despite comparable total cholesterol [14]. Subsequent studies have demonstrated that individuals with low HDL-C have a more than threefold higher risk of developing AAA [15], and each 0.4 mmol/L increase in HDL-C corresponds to a 24% reduction in odds [16]. In a recent large cross-sectional study of 1.5 million women and 0.8 million men, low HDL-C was the strongest risk factor for AAA, with each 0.37 mmol/L lower HDL-C associated with a 38% higher risk of AAA [17]. Peng et al. observed a similar effect, with a 1 mmol/L increase in HDL-C reducing the risk of AAA by 74% in men and 69% in women [18]. However, Salvador-González et al. reported that there was no difference in HDL-C levels among AAA patients and healthy controls [19].

**Table 1:** Epidemiology studies on the association between HDL and AAA

Author (Year)	Population	HDL Measurement	Outcome Assessed	Main Findings	Ref
Norrgård (1985)	Patients with AAA treated at the Surgical Clinic, University Hospital of Umeå and healthy controls; *N* = 102 (51 AAA cases)	HDL-C	Presence of AAA	HDL-C was 36.9% lower in male AAA patients and 37.8% lower in female AAA patients compared to healthy controls.	[13]
Simoni (1996)	Italian adults aged 65–75 years in AAA screening program; *N* = 1,601 (70 AAA cases)	ApoA-I, HDL-C	Presence of AAA (> 29 mm renal)	HDL-C and ApoA-I were significantly lower in AAA patients, with mean reductions of 16.3% and 12.5%, respectively.	[14]
Lindholt (2001)	Danish men with small AAA; *N* = 138	HDL-C	AAA growth rate (mm/year)	No association was found between AAA expansion and HDL-C.	[23]
Forsdahl (2009)	Norwegian adults aged 25–82 years; *N* = 4,345 (119 AAA cases); 7-year follow-up	HDL-C	Presence of AAA (≥ 35 mm renal, or ≥ 5 mm infrarenal dilatation)	Low HDL-C was strongly associated with the development of new AAA. Compared to HDL ≥ 1.83 mmol/L, those with HDL < 1.25 mmol/L had a 3.25-fold increased risk of developing AAA.	[15]
Golledge (2010)	Australian men aged 65–83 years from HIMS; *N* = 3,327 (245 AAA cases)	HDL-C	Presence of AAA (≥ 30 mm renal)	Serum HDL-C was significantly lower in AAA cases. Each 0.4 mmol/L increase in HDL-C was associated with 24% lower odds of AAA (OR, 0.76).	[16]
Ahnström (2010)	Swedish AAA patients and elderly healthy controls; *N* = 557 (343 AAA cases)	ApoA-I, ApoM	Presence of AAA	Plasma levels of ApoA-I and ApoM were significantly lower in AAA patients. ApoA-I remained significantly and inversely associated with AAA after multivariate adjustment (OR = 0.53). ApoM was not independently associated after adjustment for ApoA-I.	[20]
Takahashi (2011)	Japanese patients undergoing elective AAA surgery under 70 years of age; *N* = 101	HDL-C, LDL-C/HDL-C ratio	Postoperative CVE: stroke, CAD interventions, other vascular surgeries	Patients with later CVEs had significantly lower preoperative HDL-C and higher LDL-C/HDL-C ratio. HDL-C ≤ 35 mg/dL was associated with a 3.07-fold increased risk. No association found for triglycerides or LDL-C.	[24]
Hellenthal (2012)	Patients with AAA undergoing elective open or endovascular repair; *N* = 218 (59 small, 64 medium, 95 large AAA); 69 matched controls	HDL-C	AAA diameter (small: 30–44 mm, medium: 45–54 mm, large ≥ 55 mm).	HDL-C levels were inversely associated with aneurysm size. Serum HDL decreased from 1.14 mmol/L in small AAA to 0.80 mmol/L in large AAA.	[21]
Burillo (2015)	Spanish AAA patients; *N* = 116 (90 small AAA, 26 large AAA)	ApoA-I, HDL-C	Aortic diameter, thrombus volume	ApoA-I and HDL-C levels were significantly lower in large AAA patients. ApoA-I inversely correlated with aortic size and thrombus volume.	[22]
Burillo (2015)	Danish registry patients with AAA (*N* = 6,560) and AIOD (*N* = 23,496)	HDL-C	AAA vs. AIOD diagnosis	AAA patients had an almost two-fold lower concentration of HDL-C (0.89 mmol/L) compared with AIOD patients (1.59 mmol/L)	[22]
Burillo (2015)	Danish men with small AAA; *N* = 122	HDL-C	AAA growth rate (mm/year) and need for surgical repair	HDL-C was an independent predictor of aneurysmal growth rate. Lower HDL-C was associated with increased need for surgical repair. Patients with HDL-C below the median had significantly higher rates of surgical intervention.	[22]
Cagli (2016)	White asymptomatic AAA patients; *N* = 120	MHR	AAA diameter (mm)	Patients with higher MHR (≥ 15) had significantly larger aneurysms. MHR was significantly correlated with aneurysm size.	[28]
Salvador-González (2016)	Spanish men aged 65–74 years with AAA; *N* = 651	HDL-C	Presence of AAA (≥ 30 mm renal)	HDL-C levels were similar between groups (1.4 mmol/L in both AAA and non-AAA) with no significant association observed.	[19]
Carter (2020)	British and American adults without prior cardiovascular disease attending screening clinics; *N* = 2,331,943 (12,729 AAA cases)	HDL-C	Presence of AAA (≥ 3 cm infrarenal aortic diameter)	Lower HDL-C was inversely associated with AAA. Each 0.37 mmol/L decrease in HDL-C corresponded with a 38% higher risk of AAA.	[17]
Lin (2023)	Chinese adults from AAA screening program; *N* = 9,559 (219 AAA cases)	HDL-C, NHHR	Presence of AAA	NHHR was significantly higher in AAA patients. Compared to the low NHHR group (< 2.50), the highest NHHR group (> 3.51) had 4.2-fold higher odds of AAA. NHHR outperformed HDL-C, LDL-C, and TC in AAA risk prediction.	[27]
Koba (2023)	Japanese patients aged 40–70 years in the IPHS cohort; *N* = 95,723 (378 AAA cases)	HDL-C	Presence of AAA and AAA related death defined using ICD codes	Patients with HDL-C < 40 mg/dL had more than a double risk of death from AAA compared with those with levels ≥ 60 mg/dL.	[25]
Liu (2023)	Newly diagnosed AAA patients; *N* = 337	HDL-C; ApoA-I	Presence of AAA (> 3 cm diameter), AAA diameter, and rupture rate	HDL-C and ApoA-I were both inversely associated with AAA rupture rates. HDL-C and ApoA-I were 22.8% and 20.6% lower, respectively, in patients who experienced an AAA rupture compared to those who didn’t.	[26]
Peng (2025)	UK Biobank cohort of adults > 60 years of age; *N* = 181,866 (1,877 AAA cases)	HDL-C	Presence of AAA and AAA related death	HDL-C was inversely and dose-dependently associated with AAA risk in both sexes. For every 1 mmol/L increase in HDL-C, the risk of developing AAA decreased 74% in men and 69% in women. HDL-C was inversely associated with AAA-related death in males but was not significant in females.	[18]

HDL, high-density lipoprotein; AAA, abdominal aortic aneurysm; ApoA-I, apolipoprotein A-I; HDL-C, HDL-Cholesterol; HIMS, Health in Men Study; OR, odds ratio; ApoM, apolipoprotein M; LDL-C, low density lipoprotein cholesterol; CVE, cardiovascular event; CAD, coronary artery disease; AIOD, aortoiliac occlusive disease; MHR, monocyte-to-HDL ratio; ARIC, Atherosclerosis Risk in Communities; ICD, international classification of diseases; NHHR, non-HDL-C/HDL-C ratio; TC, total cholesterol; IPHS, Ibaraki Prefectural Health Study; HR, Hazard Ratio

Emerging research suggests that ApoA-I is a better marker for HDL than HDL-C [9]. Ahnström et al. found a statistically significant decrease in ApoA-I among AAA patients, but not in cholesterol [20]. Likewise, Simoni et al. reported a 12.5% reduction in ApoA-I among AAA patients compared to healthy controls [14].

The association of HDL with AAA progression remains inconclusive. Hellenthal et al. observed that HDL-C declined from 1.14 mmol/L in small AAAs (30–40 mm) to 0.80 mmol/L in large AAAs (≥ 55 mm) [21]. In the study of Burillo et al., lower HDL-C independently predicted faster AAA enlargement and an increased need for repair [22]. However, Lindholt et al. found no relationship between baseline HDL-C and AAA expansion [23]. Among patients undergoing elective repair, those with a preoperative HDL-C level of ≤ 35 mg/dl experienced a threefold increase in postoperative cardiovascular events [24]. Low HDL-C also appears to correlate with increased mortality. Koba et al. reported that a per-1 standard deviation increase in HDL-C, approximately 14.5 mg/dL, was associated with a 29% lower risk of aneurysm mortality [25]. In a separate study of 181,866 individuals, lower HDL-C levels were inversely associated with AAA-related death in males but not females [18]. HDL-C and ApoA-I were both inversely associated with AAA rupture rates. HDL-C and ApoA-I were 22.8% and 20.6% lower, respectively, in patients who experienced an AAA rupture compared to those who didn’t [26].

In addition to HDL-C or apoA-I concentrations, composite lipid ratios and inflammatory indices may improve AAA risk prediction. In a screening cohort of 9,559 patients, a non-HDL-C/HDL-C ratio (NHHR) greater than 3.51 was associated with a 4.2-fold higher odds of AAA compared to an NHHR less than 2.50 [27]. NHHR outperformed HDL-C, LDL-C, or total cholesterol as a predictor [27]. Furthermore, Cagli et al. reported that a monocyte-to-HDL-C ratio (MHR) greater than 15 was significantly associated with larger aortic diameters [28]. High MHR patients had higher monocyte counts and lower HDL-C [28].

## Protective Mechanisms of HDL in AAA

**Fig. 1:**
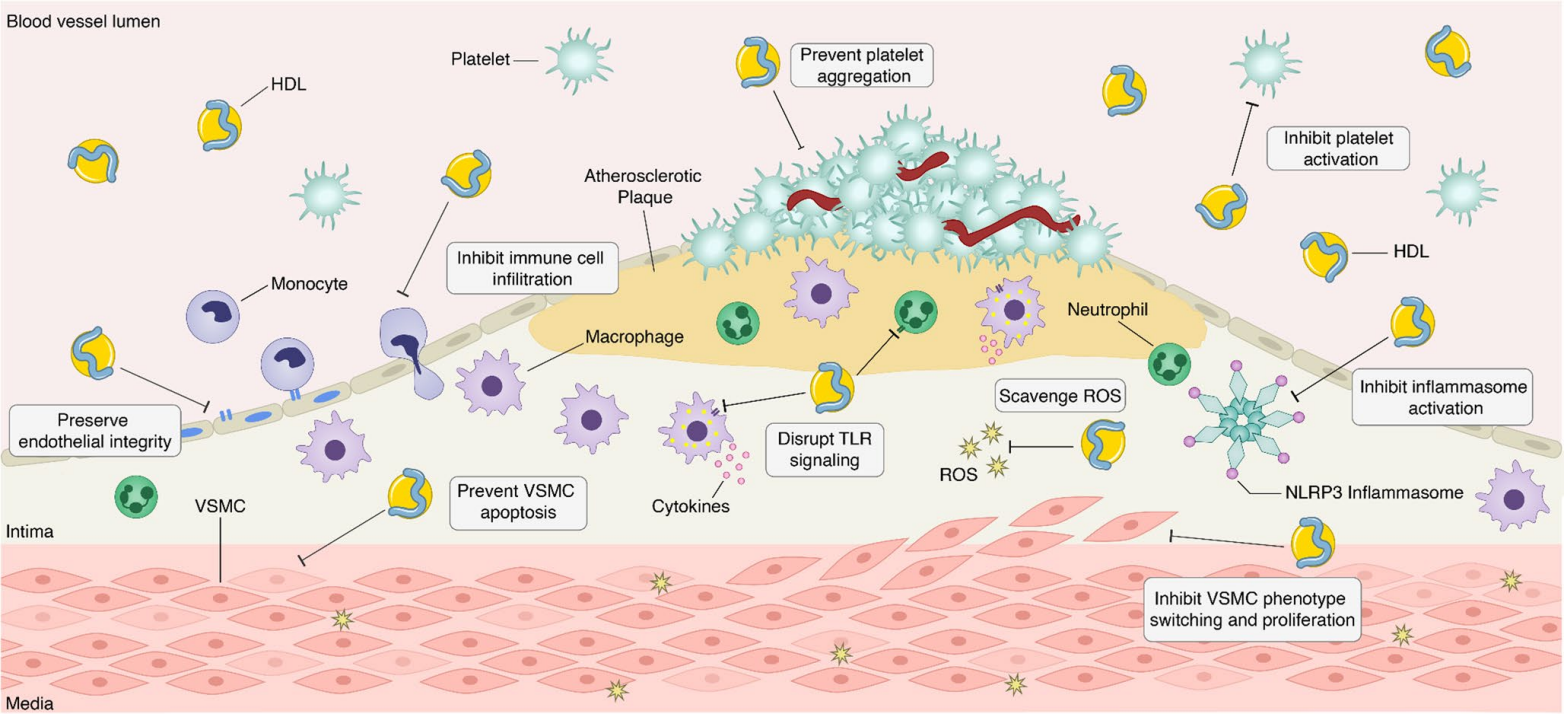
Protective effects of high-density lipoprotein (HDL) in abdominal aortic aneurysm (AAA). HDL attenuates multiple pathological processes involved in the formation of AAA. HDL preserves endothelial integrity, thereby reducing the infiltration of inflammatory cells into the aortic wall. HDL also prevents platelet activation and aggregation, diminishing thrombosis and subsequent inflammatory signaling. Upon entry into the aortic wall, HDL suppresses innate immune cell activity by inhibiting Toll-like receptor (TLR) signaling, reducing cytokine release, scavenging reactive oxygen species (ROS), and blocking NLRP3 inflammasome activation. Furthermore, HDL stabilizes vascular smooth muscle cells (VSMCs) by inhibiting apoptosis and phenotype switching

HDL has emerged as a multifaceted protective factor in AAA. Beyond its traditional role in cholesterol transport, as illustrated in Fig. 1, HDL can also protect against aneurysm development by suppressing vascular inflammation, preserving VSMC and endothelial integrity, and inhibiting thrombus formation.

### Anti-Inflammatory Effects

Although traditionally considered an end stage of atherosclerosis, AAA is now widely recognized as a chronic inflammatory disease [7, 29]. Infiltrating neutrophils and macrophages secrete matrix metalloproteinases (MMPs), which degrade elastin within the medial layer. The engagement of Toll-like receptors (TLRs) and the assembly of the NACHT, LRR and PYD domains-containing protein 3 (NLRP3) inflammasome drives the production of proinflammatory cytokines and chemokines, such as monocyte chemoattractant protein 1 (MCP-1) and interleukin-1β (IL-1β), which amplifies proteolytic and oxidative injury [7, 29, 30].

#### Innate Immune System

HDL inhibits myeloid cell recruitment by downregulating adhesion molecules and chemokine signaling. In monocyte-derived macrophages differentiated with phorbol 12-myristate 13-acetate (PMA) or macrophage colony-stimulating factor (M-CSF), both HDL and ApoA-I reduced monocytic integrin surface marker expression and spreading [31–33]. This effect was also observed in neutrophils, where HDL-mediated cholesterol efflux reduced the expression of CD11b, a surface receptor required for neutrophil extravasation [34]. In addition to its effects on integrins, HDL lowers the expression of chemokines and their receptors in monocytes, attenuating chemotactic responses [35]. This activity appears to be dependent on cholesterol efflux, as treatment with cyclodextrin, a cholesterol-depleting agent, reproduced the chemokine-lowering effects of HDL [32, 33, 36]. Iqbal et al. reported that this pathway is preserved in ABCA1-deficient cells, indicating that ABCA1 is not required for HDL-mediated inhibition of chemotaxis [36]. Other mechanisms of cholesterol efflux, including ABCG1, SR-BI, or passive diffusion, likely contribute instead.

In addition to limiting extravasation, HDL attenuates pro-inflammatory signaling through its actions on lipid rafts. Lipid rafts are dynamic, cholesterol-enriched plasma membrane microdomains where various inflammation signaling receptors, including toll-like receptors (TLR), are located [37]. In AAA, toll-like receptor 4 (TLR4) expression is upregulated, and its activation promotes MyD88-dependent inflammatory signaling that accelerates aneurysm progression [38]. Cholesterol accumulation promotes TLR4 clustering within lipid rafts, amplifying receptor dimerization and NF-κB activation. In macrophages deficient in both ABCA1 and ABCG1, elevated cholesterol concentrations in lipid rafts augment TLR4 clustering and MyD88-dependent proinflammatory signaling [39–41]. These findings were corroborated in macrophage-specific ABCA1/ABCG1 knockout mice, which exhibited increased secretion of pro-inflammatory cytokines [42]. In contrast, HDL restricts the localization of TLRs within lipid rafts through ABCA1-mediated cholesterol efflux, thereby blunting MyD88-dependent NF-κB activation and the release of pro-inflammatory cytokines [43, 44]. In macrophages stimulated with LPS, HDL significantly inhibited TLR4 expression and downstream pro-inflammatory responses [41]. Interestingly, Westerterp et al. observed that increasing HDL concentration in macrophage-specific ABCA1/ABCG1 knockout mice reduced MCP-1 secretion, despite the absence of macrophage ABCA1/ABCG1 receptors [42]. They attribute this effect to residual cholesterol efflux pathways [42].

#### Inflammasome

The NACHT, LRR and PYD domains-containing protein 3 (NLRP3) inflammasome is a multiprotein complex that promotes the maturation and release of IL-1β in response to cellular injury [45]. IL-1β signaling is implicated in AAA formation, where it facilitates leukocyte recruitment through downstream NF- κB pathways [46]. Inhibiting IL-1β signaling through NLRP3 inflammasome suppression reduced AAA in several experimental rodent models [47]. HDL has been reported to inhibit NLRP3 inflammasome activation [48, 49], suggesting a potential mechanism by which HDL may exert protective effects against AAA.

Thacker et al. first demonstrated that HDL inhibits both inflammasome activity and IL-1β secretion by preventing cholesterol crystal (CC) induced lysosomal damage [48]. CCs are insoluble, needle-like structures that, following phagocytosis, destabilize lysosomal membranes, resulting in the release of cathepsins and subsequent activation of the NLRP3 inflammasome [48, 50]. When co-treated with CCs, HDL prevented lysosomal rupture in THP-1 macrophages [48]. The mechanism by which HDL protects against CC-induced lysosomal rupture is unknown, although it may relate to its ability to limit complement 3b deposition on the crystal surface [48, 51].

Beyond CCs, HDL also affects other NLRP3 inflammasome components. In human macrophages, HDL treatment reduced the expression of *IL1β* and *NLRP3* [48]. Within 30 min of HDL treatment, decreased *IκK-α* expression was observed, suggesting suppression of NF-κB [48]. It is likely that HDL modulates NLRP3 inflammasome components through attenuated NF-κB signaling. HDL is also thought to modulate the NLRP3 inflammasome by facilitating cholesterol efflux. Cholesterol is a key regulator of inflammasome activity, and cholesterol trafficking within macrophages is a requirement for NLRP3 inflammasome activation [49, 52, 53]. Knockout of ABCA1/ABCG1 receptors in myeloid cells resulted in increased caspase-1 cleavage activity, an indicator of NLRP3 inflammasome activation [49]. NLRP3 inflammasome activation was reversed by HDL treatment in ABCA1/ABCG1 knockout mice, resulting in a nearly 50% reduction in IL-1β expression [49].

#### Complement System

HDL associates with nearly half of all known complement proteins, indicating that HDL plays a crucial role in modulating complement function [54, 55]. In the *HDL Proteome Watch*, a database of more than 250 proteins that reported to associate with HDL, complement C3, C4-b, C4-a, and C9 were the most frequently reported complement proteins [54]. Complement proteins are highly upregulated in AAA, with C3 levels increasing 125-fold in AAA [56]. HDL likely acts as a sequester of complement proteins, limiting their ability to propagate the complement cascade [55]. HDL also associates with several inhibitors of complement activity. Among the most reported HDL-associated inhibitors is clusterin (apolipoprotein J), which was detected in 45 of the 51 different HDL proteomic studies [54]. Clusterin inhibits the cytolytic activity of C5b-9, the membrane attack complex responsible for forming the pores in cell membranes that lead to cell lysis. C5b-9 depositions have been detected in AAA and are thought to contribute to pathological remodeling of the vascular wall [57, 58]. By serving as a carrier of clusterin, HDL protects against vascular and cellular damage induced by complement activation.

### VSMC Function

VSMCs are highly differentiated cells primarily responsible for maintaining the structural integrity and contractile function of the aortic wall. In AAA, VSMC dysfunction significantly contributes to aortic wall weakening and disease progression [59].

#### Phenotype Modulation

Under healthy conditions, VSMCs maintain a quiescent, contractile phenotype that supports vascular integrity [59]. However, during AAA development, VSMCs undergo phenotype switching towards a more proliferative, synthetic, and migratory state in response to pathological stimuli within the aneurysmal environment [59].

Cholesterol accumulation within VSMCs promotes dedifferentiation, characterized by the loss of contractile markers and adoption of a macrophage-like phenotype [60–62]. HDL counters cholesterol-induced phenotypic switching, restoring the expression of contractile markers. This protective effect is dependent on ABCA1, as HDL failed to reverse dedifferentiation in ABCA1-deficient VSMCs [62]. Upon binding to ABCA1, HDL normalizes the expression of microRNA-143/145 and myocardin, while repressing the transcription factor Kruppel-like factor 4 (KLF4). This promotes the re-expression of contractile proteins, thus restoring the differentiated contractile VSMC phenotype [61, 62].

The role of cholesterol removal in modulating the phenotype of VSMCs remains a topic of debate. Vengrenyuk et al. proposed that cholesterol removal is necessary, as treatment with cyclodextrin replicated the HDL-induced restoration of VSMC phenotype. However, they did not measure changes in intracellular cholesterol or any other markers of cholesterol efflux [61]. Conversely, Castiglioni et al. reported that HDL induced similar levels of cholesterol efflux in both wild-type and ABCA1-deficient VSMCs, yet the reversal of cholesterol-induced phenotypic switching occurred predominantly in wild-type cells [62]. These findings suggest that cholesterol efflux alone may be insufficient to fully explain HDL’s phenotype modulating effect. It is possible that ABCA1-dependent signaling pathways could contribute independently of, or synergistically with, cholesterol efflux. Further research is needed to confirm the extent to which cholesterol efflux modulates the VSMC phenotype.

#### Apoptosis

VSMC loss in AAA is largely driven by mitochondrial dysfunction and apoptosis resulting from oxidative stress and pro-inflammatory cytokines [59]. Recent mechanistic studies have demonstrated that HDL exerts robust anti-apoptotic effects in VSMCs.

Mitochondrial dysfunction is a hallmark of VSMC apoptosis in AAA. During AAA development, Ang II induces mitochondrial fragmentation by upregulating dynamin-related protein 1 (DRP1) [63]. This is accompanied by loss of mitochondrial DNA (mtDNA), reduced expression of electron transport chain (ETC) proteins, impaired oxidative phosphorylation, and enhanced ROS generation, ultimately leading to apoptosis [63]. In murine aortic smooth muscle cells, sHDL administration prevented Ang II-induced DRP1 upregulation and subsequent mitochondrial fragmentation [63]. Further restoration of mitochondrial function by HDL is evidence by preservation of mtDNA content, ETC protein expression, and oxygen consumption rates in both Ang II and H_2_O_2_-exposed VSMCs [63].

HDL preserves mitochondrial function by attenuating ROS-driven injury. HDL scavenges and neutralizes lipid hydroperoxides (LOOH) from oxidized lipoproteins and cell membranes, functioning as a “sink” for reactive lipid species [64]. These reactive lipid species are then transported to the liver via HDL to undergo hepatic catabolism [65]. This antioxidative capacity is partly dependent on ApoA-I, which reduces cholesteryl ester hydroperoxides and phospholipid hydroperoxides via the oxidation of methionine residues [66, 67]. Apolipoprotein A-IV (ApoA-IV) also contributes to HDL’s antioxidative capacity by inhibiting lipoprotein oxidation [68]. The antioxidative activity of HDL is further enhanced by HDL-associated enzymes such as paraoxonase 1 (PON1), peroxiredoxin-6 (PRDX6), and platelet-activating factor acetylhydrolase (PAF-AH), which hydrolyze oxidized lipids [69–71].

HDL has also been reported to directly modulate mitochondrial function [72]. In ApoA-I-deficient mice, the cardiac mitochondrial Coenzyme Q (CoQ) pool is reduced, leading to impaired electron transfer from Complex II to Complex III and enhanced ROS generation [73]. Restoration of CoQ levels rescues mitochondrial respiration, indicating that ApoA-I preserves mitochondrial function in part by maintaining an adequate CoQ pool and coupling between Complex II and Complex III [73, 74]. Although these observations derive from ischemia-reperfusion models, they provide a mechanistic precedent of ApoA-I dependent maintenance of the mitochondrial CoQ pool and Complex II to Complex III coupling. In the context of AAA, HDL could similarly preserve ETC function by preserving CoQ content and inhibiting mitochondrial injury in VSMCs.

HDL also regulates mitochondrial function through sphingosine-1-phosphate (S1P), one of the more than 200 individual molecular lipid species that associates with HDL [75]. HDL-bound S1P activates pro-survival kinase pathways, including the RISK (Akt/ERK1/2) and SAFE (JAK/STAT3) pathways [72, 76]. These pathways stabilize mitochondrial membrane potential, preventing cytochrome c leakage. As a result, caspace-3 activation decreases, and the mitochondrial permeability transition pore is less likely to open during stress, preserving mitochondrial function [72].

HDL inhibition of VSMC apoptosis is further enhanced by the presence of α1-antitrypsin (AAT), an endogenous potent inhibitor of neutrophil elastase [77]. Elastase, which is abundant in AAA, degrades ECM proteins and induces VSMC anoikis, the loss of VSMCs due to ECM detachment [7]. HDL selectively binds AAT and inhibits elastase activity [77]. In human VSMCs exposed to AAA thrombus conditioned media, AAT-enriched HDL preserved cell adhesion and viability by blocking elastase-induced detachment and apoptosis [77].

### Endothelial Protection

Although most studies on AAA have focused on VSMC loss and ECM degradation, increasing evidence suggests endothelial dysfunction is an important early event in AAA pathogenesis [78]. Aneurysm formation is thought to initiate upon endothelial dysfunction, where environmental insults, such as smoking and chronic inflammation, increase endothelial permeability and upregulate adhesion molecules that facilitate leukocyte infiltration [78]. HDL is well established as a key modulator of endothelial function [79]. A substantial component of this vasoprotective activity is mediated by HDL-associated S1P, which signals through endothelial S1P receptors to maintain endothelial integrity and reduce vascular inflammation [80].

HDL has previously been shown to simulate endothelial nitric oxide synthase (eNOS) production and recoupling [81, 82], suggesting a potential endothelial protective mechanism of HDL in AAA. eNOS is responsible for the synthesis of nitric oxide (NO), a potent vasodilator and anti-inflammatory mediator [83]. Within endothelial cells, eNOS is localized to lipid rafts. Cholesterol depletion disrupts this localization, redistributing eNOS and reducing NO production [84]. Binding of HDL to SR-BI elevates intracellular ceramide through an Akt-independent process, which facilitates eNOS activation [85]. In parallel, SR-BI binding activates Src family kinases upstream of P13K-Akt, leading to eNOS phosphorylation and enhanced NO production [86]. HDL-bound S1P further amplifies this response by activating S1PR1/3 and downstream P13K-Akt signaling, promoting eNOS phosphorylation and NO generation [87]. The resulting NO increase induces vasodilation and, via p38MAPK/CREB-mediated transcriptional feedback, upregulates eNOS to sustain NO production [86, 88].

Several studies have demonstrated HDL’s ability to inhibit the expression of endothelial surface adhesion molecule expression. In human umbilical vein endothelial cells (HUVEC), plasma-derived HDL dose-dependently inhibited TNF-α-induced VCAM-1, ICAM-1, and E-selectin expression [89, 90]. Interestingly, further studies were unable to replicate this effect in more physiologically relevant cell types, such as coronary artery and aortic endothelial cells [91, 92]. However, the ability of HDL to reduce endothelial surface adhesion molecule expression was demonstrated in several in vivo studies [93–95]. In a porcine model of inflammation, administration of reconstituted HDL reduced E-selection expression by approximately 76% [93]. In vivo, HDL treatment also reduced VCAM-1 [94, 95] and ICAM-1 expression [95].

### Anti-Thrombotic Effects

More than 75% of AAA patients present with a non-occlusive ILT [96], and greater ILT burden is associated with accelerated aneurysm growth and a higher risk of rupture [97, 98]. HDL has emerged as a potent anti-thrombotic factor [99], capable of interfering with platelet activation, adhesion, and aggregation.

HDL disrupts key steps in platelet activation by modulating membrane cholesterol and receptor clustering. Platelet activation promotes lipid raft clustering, concentrating signaling receptors and enhancing integrin αIIbβ3 and granule secretion [99]. Via cholesterol efflux, HDL disrupts platelet lipid raft clustering and reduces the surface expression of FcγRII and glycoprotein Ib (GpIb) [99]. This suppresses platelet responsiveness and interferes with the GpIb-V-IX signaling complex.

HDL also acts at later stages of thrombus development by limiting platelet adhesion and aggregation. HDL binding to von Willebrand factor (VWF) prevents platelet self-association, thereby reducing platelet adhesion and creating a less thrombogenic environment [100]. HDL also interferes with platelet aggregation by modulating the fibrinogen receptor integrin αIIbβ3. HDL inhibits thrombin-induced fibrinogen binding and platelet aggregation by suppressing phosphatidylinositol 4,5-bis-phosphate (PIP2) formation, a secondary messenger required for αIIbβ3 activation [101]. Consistent with these observations, ApoA-I infusions in a mouse model of carotid artery thrombosis inhibited platelet aggregation [102]. Additionally, apoA-IV can directly bind αIIbβ3 and inhibit platelet aggregation [103]. While only a small fraction of ApoA-IV is associated with HDL [12], it is possible that this mechanism contributes to HDL’s antithrombotic effects.

### Disentangling Anti-AAA Functions of HDL from its Anti-Atherosclerotic Effects

HDL has long been recognized for its protective effects against atherosclerosis due to its plethora protective mechanisms including mediating cholesterol efflux, regulating inflammatory responses, reducing oxidative stress, and modulating endothelial functions. With the significant overlap between anti-atherosclerosis and anti-AAA mechanisms of HDL, as well as the frat that atherosclerosis is one of strongest risk factors for AAA, it raises the question whether the anti-AAA effects of HDL are secondary to its anti-atherosclerosis effects, or both effects arise from addressing shared pathological pathway such as inflammation and endothelial dysfunction underlying these two conditions.

Answering this question firstly requires the examination of causal relationship between atherosclerosis and AAA. The prevalence of atherosclerotic lesions in AAA site, as well as the well-established correlation between atherosclerosis and AAA, had promoted the hypothesis that AAA may be caused by atherosclerosis. However, accumulating evidence suggested that atherosclerosis and AAA are separate diseases which can develop in parallel with overlapping risk factors and pathological features. The detailed discussion of their interrelationship is out of the scope of this review and is thoroughly addressed in [104, 105]. Under this framework, the anti-AAA effects of HDL may not be contingent to its anti-atherosclerosis effects, but stem from its overall cardioprotective functions.

Recent animal studies also suggested atherosclerosis-independent anti-AAA effects of HDL. Torsney et al. reported that HDL treatment reduced AAA formation on CaCl_2_-induced normocholesterolemic AAA model [106]. Our recent studies also demonstrate that HDL treatment attenuates mitochondrial loss and AAA development in the PPE (porcine pancreatic elastase) model, another well-established AAA animal model independent of hypercholesterolemia [63]. On the hypercholesterolemic AAA model, HDL treatment also reduced AAA formation at a dose not sufficient to reduce plaque area [63]. Overall, the results suggested that the anti-AAA effects of HDL were independent of hypercholesterolemia and atherosclerotic plague reduction in these animal models. Although AAA and atherosclerosis share common risk factors, standard anti-atherosclerotic therapies offer limited benefit in slowing AAA growth and lack solid evidence for preventing rupture or dissection in AAA patients [107–110]. Future large randomized clinical studies separating AAA patients based on coexisting atherosclerosis status would be helpful to better understand the AAA-specific role of HDL.

### Structural and Compositional Determinants of HDL Protective Effects

**Fig. 2:**
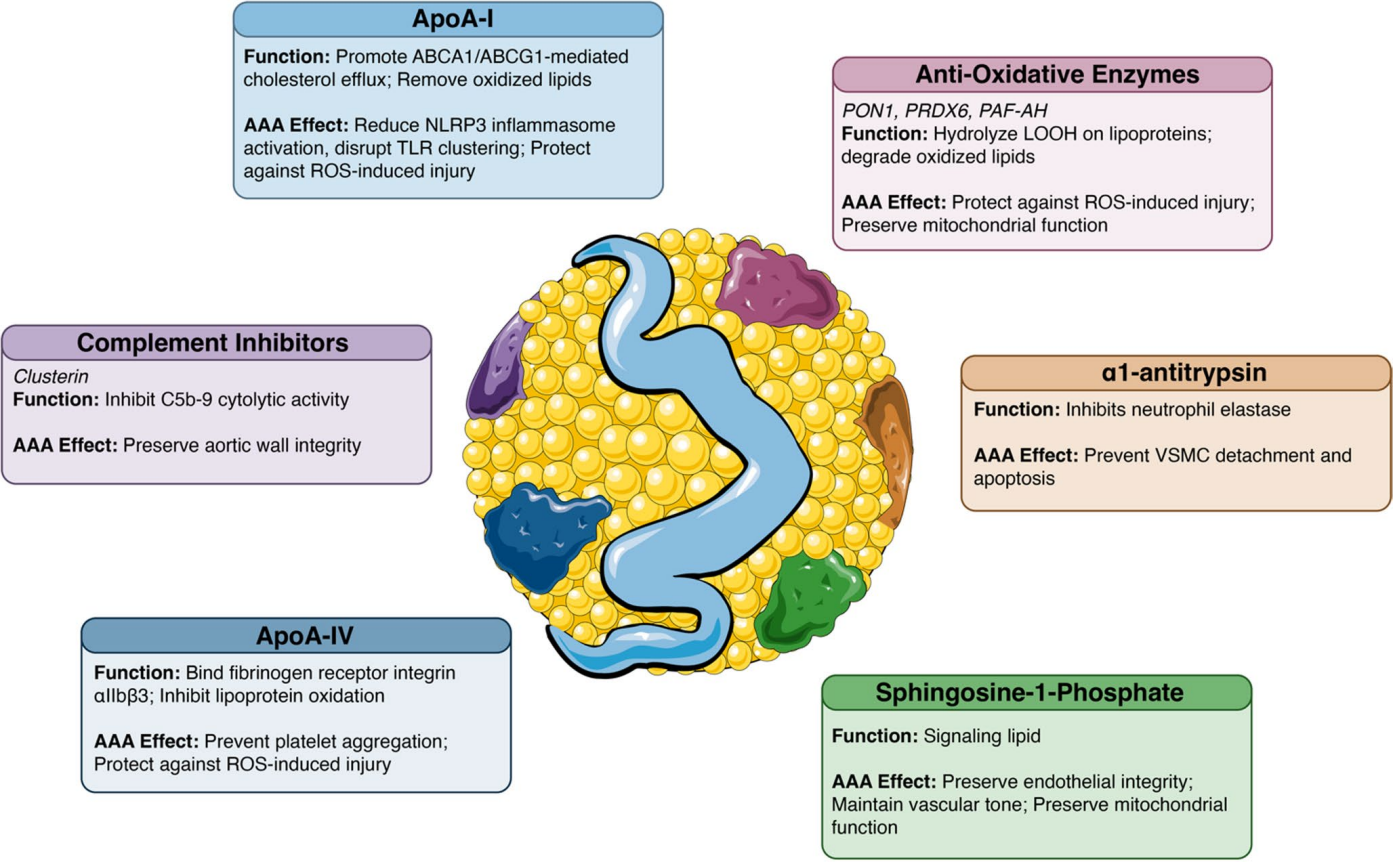
High-density lipoprotein (HDL) associated components and their proposed protective roles in abdominal aortic aneurysm (AAA)

HDL is a heterogeneous population of particles that differ in size, density, and proteomic/lipidomic composition. Epidemiological and clinical studies suggest that HDL subpopulations are a better predictor of cardiovascular risk than HDL-C alone [111]. Specifically, an increase in small, dense HDL3 is associated with worse cardiovascular outcomes, whereas higher concentrations of HDL2 are linked to a reduced cardiovascular risk [112]. However, the specific relationship between HDL subpopulations and AAA risk has not yet been clearly defined and remains an important area for future study. From component perspective, HDL particles contain hundreds of species of protein, lipid, and other bioactive molecules such as microRNAs, all of which directly affect functions of HDL. Some key HDL components and their impacts on HDL effects discussed in this section are summarized in Fig. 2. A comprehensive discussion on composition-functionality relationship is beyond the scope of this review and has been thoroughly reviewed elsewhere [111, 113].

## Dysfunctional HDL in AAA

Dysfunctional HDL encompasses a broad array of structural and functional alterations that undermine its protective function. The chronic inflammatory, proteolytic, and oxidative environment of AAA promotes the formation of dysfunctional HDL. Analysis of patient cohorts has consistently documented shifts in HDL composition (Table 2).

**Table 2:** Changes in HDL composition and functionality in AAA

Author (Year)	Subjects	HDL Changes	Ref
Ortiz-Muñoz (2009)	13 AAA patients and 23 matched controls	ApoA-I ↓ AAT ↓ Antielastase activity ↓	[77]
Delbosc (2013)	20 AAA patients and 20 matched controls	ApoA-I ↓ Small HDL ↓ Antioxidative activity ↓ Oxidized HDL ↑	[115]
Burillo (2016)	14 AAA patients and 7 matched controls	PRDX6 ↑ PON1 ↑ SAA ↑ A2M ↓ C4BP ↓	[69]
Martínez-López (2019)	41 AAA patients and 41 matched controls	ApoA-I ↓ Preβ-HDL ↓ CEC ↓	[116]
Rodríguez-Carrio (2021)	427 AAA cases and 139 matched controls	IgG anti-HDL MDA antibodies ↑	[124]
Adorni (2022)	30 AAA cases and 21 matched non-AAA atherosclerosis controls	Preβ-HDL ↓ ABCG1-CEC ↓ ABCA1-CEC ↑ LCAT activity ↑ CETP activity ↑	[117]

HDL, high-density lipoprotein; AAA, abdominal aortic aneurysm; ApoA-I, apolipoprotein A-I; AAT, alpha-1 antitrypsin; PRDX6, peroxiredoxin 6; PON1, paraoxonase-1; SAA, serum amyloid A; A2M, alpha-2-macroglobulin; C4BP, C4b-binding protein; CEC, cholesterol efflux capacity; IgG, immunoglobulin G; MDA, malondialdehyde; ABCG1, ATP-binding cassette subfamily G member 1; ABCA1, ATP-binding cassette subfamily A member 1; LCAT, lecithin-cholesterol acyltransferase; CETP, cholesteryl ester transfer protein

AAA lesions are rich in ROS generated by neutrophils, macrophages, and activated VSMCs. Myeloperoxidase (MPO), abundantly expressed in AAA tissue, catalyzes the oxidation of ApoA-I at tyrosine and methionine residues. These oxidative modifications alter the ɑ-helical domains necessary for lipid binding and interactions with ABCA1, reducing cholesterol efflux efficiency [114]. In AAA, oxidation constitutes more than 70% of all post-translational modifications of HDL, with ApoA-I containing the most oxidation-induced changes [115, 116]. AAA patients exhibit reduced HDL antioxidative capacity, likely due to oxidation-induced structural modifications [115].

Excessive enzymatic activity in AAA drives HDL lipid remodeling, shifting from lipid-poor, nascent HDL towards mature, cholesteryl ester-rich HDL. Adorni et al. demonstrated that AAA patients exhibit elevated LCAT and CETP activity [117]. This enzymatic coupling accelerates the maturation of nascent HDL via LCAT-mediated cholesterol esterification, while simultaneously transferring cholesteryl esters to ApoB lipoproteins via CETP, depleting the pool of spherical, phospholipid-rich HDL favored by ABCG1-mediated efflux. In AAA patients, ABCG1-CEC is reduced by 16% whereas ABCA1-CEC increased by 32% [117]. This likely reflects compensatory cholesterol efflux through lipid-poor nascent particles as mature HDL function declines. Preβ-HDL levels are reduced nearly threefold, implying that the ABCA1-CEC increase is due to greater ApoA-I exposure on remodeled HDL rather than expansion of the nascent HDL pool [117].

The aneurysmal environment is characterized by elevated proteolytic activity, with neutrophil elastase and MMPs acting on both ECM components and circulating lipoproteins, such as HDL. Proteolytic cleavage of ApoA-I by neutrophil elastase generates elastase-digested HDL with altered structural integrity [118]. These particles exhibit an increased binding affinity for macrophages and are internalized and degraded via SR-BI at an approximately threefold higher rate than native HDL [118]. This suggests that proteolytic remodeling accelerates HDL catabolism, thereby reducing the availability of functional HDL. Proteolysis also impairs the anti-proteolytic capacity of HDL by depleting AAT. As discussed previously, HDL-bound AAT has been shown to prevent elastase-mediated ECM degradation, VSMC detachment, and apoptosis [77]. Moreover, HDL in AAA can become a carrier for active proteases. MMP-9 has been detected in association with HDL and can impair HDL function [119].

In addition to oxidation and proteolysis, AAA HDL undergoes carbamylation and glycation. Carbamylation, mediated by MPO-derived cyanate, modifies lysine residues on ApoA-I, impairing receptor binding and lipid interactions [120]. In vascular tissue, carbamylated HDL has been linked to pro-inflammatory signaling, including upregulation of endothelial adhesion molecules [121]. Glycation generates advanced glycation end products that disrupt the conformation of HDL and promote its clearance [122]. IgG autoantibodies against both native [123] and malondialdehyde (MDA)-modified HDL [124] have been detected in AAA patients, suggesting that these modifications create neoepitopes that trigger immune-mediated HDL removal.

Chronic inflammation induces HDL remodeling. Serum amyloid A (SAA), an acute-phase protein, can displace ApoA-I during periods of elevated inflammation [55, 125]. SAA-rich HDL exhibits reduced cholesterol efflux capacity and increased pro-inflammatory signaling [126]. In AAA patients, HDL contains significantly more SAA than healthy controls [22]. This enrichment may be driven by sustained acute-phase responses triggered by systemic cytokines, as well as local production of SAA by vascular and immune cells.

## HDL Therapy

Given the pleiotropic effects of HDL, there is strong interest in the development of HDL therapeutics. Reconstituted HDL (rHDL) and synthetic HDL (sHDL) have emerged as promising therapeutic strategies for mimicking the cardioprotective effects of native HDL. These particles resemble discoidal HDL, consisting of phospholipids formulated with either full-length, purified ApoA-I (rHDL) or smaller ApoA-I mimetic peptides (sHDL) [127]. Several clinical trials have demonstrated that rHDL/sHDL treatment is effective at increasing both HDL levels and functionality [128, 129], suggesting that HDL therapies could be a viable treatment for AAA.

Torsney et al. first demonstrated that elevating HDL concentration is a potential therapeutic strategy for inhibiting the development of AAA. In an angiotensin (ang) II-induced hypercholesterolemic mouse model, pretreatment with rHDL prevented aneurysm formation, with the mean aortic diameter reduced to 1.41 mm, compared to 2.29 mm in controls [106]. Co-treatment with rHDL also significantly attenuated aneurysm growth, reducing the mean aortic diameter to 1.45 mm from 2.29 mm in controls. Aneurysm and rupture incidence was reduced to 37.5–40% in the co-treatment groups and to 33% with pretreatment, although rHDL treatment did not alter rupture-related mortality [106]. rHDL treatment approximately doubled plasma HDL concentration but had no effect on body weight, systolic blood pressure, or atherosclerotic plaque burden, suggesting that the protective effects were specific to aneurysm suppression rather than systemic lipid lowering [106]. Similarly, intraperitoneal administration of 4-F, an ApoA-I mimetic peptide, reduced aortic dilation [22].

Building on these studies, Liu et al. investigated the effects of sHDL in several murine models of AAA. In a PCSk9/Ang II-induced AAA mouse model, administration of sHDL decreased AAA incidence by more than 30% and reduced the maximum aortic diameter [63]. sHDL treatment also led to reduced plasma levels of triglycerides and total cholesterol, inhibited macrophage infiltration, and preserved aortic elastin integrity [63]. The protective effects of sHDL were further validated in a murine porcine pancreatic elastase (PPE) model, which is not dependent on hyperlipidemia. sHDL treatment significantly reduced aortic diameter in both male and female mice, demonstrating a protective role for sHDL that is independent of changes to plasma lipid levels [63].

In contrast, Delbosc et al. reported that administration of HDL isolated from healthy patients did not reduce aortic diameter or AAA incidence in a PPE-induced rat AAA model [130]. There are several possible explanations for this discrepancy. There were substantial differences in dosing regimens. Liu et al. administered 30 mg/kg of sHDL three times a week for four weeks [63], whereas Delbosc et al. administered 6 mg/kg of HDL once a week for two weeks [130]. Given that the plasma half-life of ApoA-I following HDL administration ranges between 6 and 24 h [131], the dosing regimen used by Liu et al. likely resulted in prolonged therapeutic exposure compared to that of Delbosc et al. Furthermore, the composition and structure of HDL are critical determinants of efficacy. The discoidal sHDL formulation used by Liu et al. is optimized for cholesterol efflux via ABCA1, whereas spherical HDL is less effective in promoting cholesterol efflux [132]. Moreover, donor-derived HDL is susceptible to compositional variability and may be functionally heterogeneous [133]. It is likely that the combination of differences in dosing, particle structure, and functional quality of HDL contributed to the lack of efficacy observed by Delbosc et al.

Beyond exogenous HDL infusion, pharmacological agents have been investigated in AAA for their ability to enhance HDL levels. Fenofibrate, a peroxisome proliferator-activated receptor alpha (PPARα) agonist, can raise serum HDL-C by more than 10% [134]. Fenofibrate treatment significantly reduced aortic expansion in both Ang-II-induced ApoE^−/−^ and Ldlr^−/−^ mouse models [135, 136]. Although fenofibrate did not significantly reduce AAA growth in the FAME-2 placebo-controlled clinical trial [137], the minimal aneurysm progression observed in the control group over the short period likely limited the ability to detect treatment effects, underscoring the need for longer-term, larger-scale studies to properly evaluate the impact of HDL elevation on AAA progression and rupture risk.

While there is ample evidence that HDL therapies significantly attenuate AAA progression in animal models [22, 63, 106], currently there is no HDL therapy in clinical development for AAA. Some rHDL products, such as CER-001 and CSL112, have been tested for atherosclerotic diseases [138]. However, the failure of rHDL to reduce the risk of recurrent cardiovascular events in a large Phase 3 clinical trial [139] suggested that simply raising HDL levels is not sufficient. Indeed, growing evidence suggests that HDL functionality, rather than HDL levels, is a more relevant predictor of clinical benefit. Thus, future HDL-based therapies for AAA would require a shift from elevating HDL levels to enhancing its AAA-relevant protective functions based on understanding of AAA pathology, protective mechanisms of HDL, and structure/composition-function relationship of HDLs.

## Conclusion

Abdominal aortic aneurysm is a life-threatening vascular condition for which no FDA-approved pharmacological therapies currently exist. Epidemiological studies consistently link low HDL levels with increased AAA risk, highlighting HDL as a potential therapeutic target. Through its anti-inflammatory, vasoprotective, anti-proteolytic, and anti-thrombotic functions, HDL contributes to maintaining aortic wall integrity. The anti-inflammatory, vasoprotective, anti-proteolytic, and anti-thrombotic properties of HDL collectively contribute to the preservation of aortic wall integrity. The emerging field of HDL therapeutics, including reconstituted and synthetic HDL formulations, holds promise for translating these protective mechanisms into effective clinical interventions. Nevertheless, further research is needed to fully elucidate the complex interplay between HDL functionality and AAA pathophysiology, as well as to optimize HDL-based treatment strategies.

## Key References

- Adorni MP, Palumbo M, Marchi C, Zimetti F, Ossoli A, Turri M, et al. HDL metabolism and functions impacting on cell cholesterol homeostasis are specifically altered in patients with abdominal aortic aneurysm. Front Immunol. 2022;13:935241. 10.3389/fimmu.2022.935241.
  - ○ This study showed that low HDL-C significantly increased the risk of mortality from aortic aneurysm over 26 years of follow-up. The findings emphasize that reduced HDL levels contribute to aneurysm-related death independent of other lipids, highlighting HDL as a protective factor in AAA.
- Gibson CM, Duffy D, Korjian S, Bahit MC, Chi G, Alexander JH, et al. Apolipoprotein A1 Infusions and Cardiovascular Outcomes after Acute Myocardial Infarction. N Engl J Med. 2024;390(17):1560-71. 10.1056/NEJMoa2400969.
  - ○ This phase 3 clinical trial tested reconstituted HDL infusions in >18,000 post myocardial infarction patients, showing rapid increases in cholesterol efflux capacity but no reduction in major adverse cardiovascular events at 90 days.
- Koba A, Yamagishi K, Sairenchi T, Noda H, Irie F, Takizawa N, et al. Risk Factors for Mortality From Aortic Aneurysm and Dissection: Results From a 26-Year Follow-Up of a Community-Based Population. J Am Heart Assoc. 2023;12(8):e027045. 10.1161/JAHA.122.027045.
  - ○ This study showed that low HDL-C significantly increased the risk of mortality from aortic aneurysm over 26 years of follow-up. The findings emphasize that reduced HDL levels contribute to aneurysm-related death independent of other lipids, highlighting HDL as a protective factor in AAA.
- Liu Y, Yu M, Wang H, Dorsey KH, Cheng Y, Zhao Y, et al. Restoring Vascular Smooth Muscle Cell Mitochondrial Function Attenuates Abdominal Aortic Aneurysm in Mice. Arterioscler Thromb Vasc Biol. 2025;45(4):523-40. 10.1161/ATVBAHA.124.321730.
  - ○ Preclinical study demonstrating that restoration of VSMC mitochondrial function via sHDL treatment reduces oxidative stress, preserves contractile phenotype, and limits AAA progression in experimental AAA mouse models.
- von Eckardstein A, Nordestgaard BG, Remaley AT, Catapano AL. High-density lipoprotein revisited: biological functions and clinical relevance. Eur Heart J. 2023;44(16):1394-407. 10.1093/eurheartj/ehac605.
  - ○ A comprehensive review of HDL biology, highlighting pleiotropic vascular functions and therapetuic strategies.

## Data Availability

No datasets were generated or analysed during the current study.
